# Liver Fibrosis progression using Fibroscan in HIV/HCV coinfected patients with undetectable HIV viral load

**DOI:** 10.7448/IAS.17.4.19636

**Published:** 2014-11-02

**Authors:** Laura Perez-Martinez, Isabel Sanjoaquin, Maria Rivero, Desiré Gil-Pérez, Santiago Letona, Carmen Irigoyen Olaiz, Piedad Arazo, Jose Ramón Blanco

**Affiliations:** 1 Infectious Diseases Department, Hospital San Pedro, Center for Biomedical Research of La Rioja (CIBIR), Logrono, Spain; 2 Infectious Diseases, Hospital Clínico Universitario Lozano Blesa, Zaragoza, Spain; 3 Infectious Diseases, Hospital de Navarra, Pamplona, Spain; 4 Infectious Diseases, Hospital Universitario Miguel Servet, Zaragoza, Spain

## Abstract

**Introduction:**

Several factors such as duration of infection, age, male gender, consumption of alcohol, HIV infection and low CD4 count have been associated with fibrosis progression rate. However, it is relatively scarce, the knowledge about the liver fibrosis progression rate in HIV-infected patients with undetectable HIV viral load (VL). For this reason, we performed the present study.

**Materials and Methods:**

Observational and multicenter study (2008–2012) conducted in four hospitals of the northern Spain. HIV/HCV (hepatitis c) virus coinfected patients ≥18 years on stable combination antiretroviral therapy (cART) (≥6 months) and with a HIV VL <50 copies/mL were selected to analyze their liver fibrosis progression. Fibrosis progression was assessed using a Fibroscan^®^ (502 STEP 3 model) and measuring a basal test and a second one at least 12 months apart from baseline. This evolution was compared with different variables such as duration of HIV/HCV coinfection, gender, age, previous treatment for HCV, HCV genotype, CD4 lymphocyte counts and the cART employed at the basal test.

**Results:**

A total of 608 patients were included (median age 29.4 years, 71.7% men). Of these, 463 patients met the inclusion criteria. In these patients, the liver fibrosis progression was nearly flat and the only variables related to a higher liver fibrosis progression were the increasing age of the patients (p=0.02) and the duration of the coinfection (p=0.001). CD4 lymphocyte counts showed a tendency to improved liver fibrosis (p=0.056).

**Conclusions:**

In HIV/HCV coinfected patients on stable cART and HIV undetectable VL, the increase in liver fibrosis rate progression was nearly flat, although it was significantly associated with the duration of the coinfection and the age of the patient. The beneficial effects of the cART were independent of the antiretroviral drug employed. A tendency to a lower fibrosis progression was observed in those patients with a higher CD4 count.

